# Internet use and depressive symptoms among Chinese older adults: Two sides of internet use

**DOI:** 10.3389/fpubh.2023.1149872

**Published:** 2023-03-09

**Authors:** Aruhan Mu, Shanshan Yuan, Zhiyong Liu

**Affiliations:** School of Medicine and Health Management, Huazhong University of Science and Technology, Wuhan, Hubei, China

**Keywords:** dark side of information technology use, internet use, depressive symptoms, aged, China, negative results

## Abstract

**Objective:**

To explore the relationship between internet use which includes time spent on the internet, internet skills, types of online activities, and depressive symptoms in older adults.

**Methods:**

We used 2020 China Family Panel Studies (CFPS) data with 3,171 older adults aged 60 years. Depression symptoms were measured with the Center for Epidemiologic Studies Depression (CES-D), and internet use was measured by time spent on the internet, internet skills, and types of online activities. Multiple linear regression models were used to explore the relationship between internet use and depressive symptoms in older adults.

**Results:**

Longer time spent on the internet was associated with higher scores of depressive symptoms (β = 0.14). Higher internet skills were associated with lower scores of depressive symptoms (β = −0.42). Watching short-form videos (β = 1.34) was associated with higher depressive symptom scores, and the use of the WeChat function (β = −0.96) was associated with lower depressive symptom scores, while online games and online shopping were not significant.

**Conclusion:**

The effect of internet use on depressive symptoms in older adults is a double-edged sword. Controlling the time spent on the internet, improving internet skills, and guiding specific a type of online activities in older adults can improve depressive symptoms through rational use.

## 1. Introduction

Depression is a major threat to the mental health of older adults with an estimated prevalence of 4–9% worldwide (1), and the prevalence of depression among people aged 60 years and older in China is the highest of all ages groups, at 20.6 to 30.7% (2). With the deepening of aging and accelerated social transformation, the mental health pressure in older adults will be even greater, with a potentially large number of older adults with moderate to high levels of depressive symptoms (3). Besides, depression is associated with substantial morbidity and mortality due to potential underdiagnosis and under-treatment (1). A study showed that depression may exacerbate the clinical manifestations of combined symptoms such as diabetes or hypertension and is a risk factor for poor outcomes of these conditions (2). Thus, more attention should be paid to the depressive symptoms of older adults.

With the development of information technology and the improvement of residents' living standards, more and more older adults use the Internet. As of June 2022, the size of internet users aged 60 and above in China reached 119 million (4). According to research data, about half of the middle-aged and older adults spends more than four hours a day online (5). Online activities have become rich and diverse, with instant messaging, short-form videos, online games, and online shopping becoming frequently used activities by older adults (5). In addition, Internet skills respond to the ability to use the Internet. People with stronger internet skills report greater willingness to adopt new technologies (5) as well as benefit from the Internet.

Evidence of the relationship between internet use and depressive symptoms in older adults is mixed. Some research found that internet use in older adults was associated with reducing depressive symptoms (6). Potential mechanisms for this association include providing a sense of self-esteem and control, maintaining and enhancing social relationships, providing leisure activities, and reducing feelings of isolation (7–11). The use of the internet by older adults in their daily lives provides new ideas for improving their depressive symptoms (7–9). However, Lifshitz et al. (12) found major online activities common among older adults, such as: information gathering, interpersonal communication, and leisure, were not associated with depressive symptoms. Moreover, Nie et al. (13) found that internet use among older adults in China was associated with higher depressive symptoms. Likewise, comparisons of internet users with others displayed a significantly higher level of depressive symptoms and anxiety among the former (14).

These contrasting findings can be explained by the macroscopic approach applied in many studies that have explored the association between internet use and depressive symptoms in older adults. On the one hand, Previous studies have used only a single indicator of whether or not they use the Internet to measure older adults' online behavior, ignoring older adults' rich internet use practices. On the other hand, Previous studies have not considered the stage-specific characteristics of Internet-generated benefits, i.e., internet use progressively generates benefits from internet access, time spent on the internet, internet skills, and types of online activities. When considering again the effect of internet use activities on depressive symptoms, the time spent on the internet and the internet skills of the sample need to be controlled.

Therefore, to remedy the shortcomings of these studies and to correctly reveal the relationship between internet use and depressive symptoms among older adults in China, time spent on the internet and internet skills should be considered, and on this basis, types of online activities in improving depressive symptoms should be examined. In this study, we conducted a data reanalysis based on the China Family Panel Studies (CFPS) data to explore the relationship between internet use and depressive symptoms in older adults, intending to provide an empirical basis for practices to improve depressive symptoms in older adults.

## 2. Methods

### 2.1. Sample and data collection

We performed a cross-sectional, secondary analysis using the data obtained from Wave 5 in 2020 of the China Family Panel Studies (CFPS), which is a national longitudinal survey started in 2010 by the Institute of Social Science Survey (ISSS) at Peking University (15). The CFPS collects individual (separate for children and adults), household, and community-level data through personal interviews (data recorded through computer-assisted personal interviewing techniques) for 25 provinces in China, which cover 95% of China's population. The CFPS interviewed a total of 14,960 households and 42,590 individuals, with a sample of individuals followed every two years.

The sample selection criteria and steps of this study: (1) Extraction of a nationally representative resample of 18,783. (2) There were 4,561 samples of older adults aged ≥60 years; (3) Missing samples of the Center for Epidemiologic Studies Depression Scale (CES-D) scores were excluded, and a final sample of 3171 was obtained.

### 2.2. Variables

#### 2.2.1. Depressive symptoms

The eight-item Center for Epidemiologic Studies Depression Scale (CES-D) was used in CFPS to examine depressive symptoms, which is widely used in China (16) and has shown good validity and reliability (Cronbach's α = 0.815) (17). In CFPS, respondents were asked to answer eight questions, namely, “I am in a low spirit” “I find it difficult to do anything” “I cannot sleep well” “I feel happy” “I feel lonely” “I have a happy life” “I feel sad” and “I feel that I cannot continue with my life” including two positive emotions items and six depression items. Response options for each are “Never” (equating to a score of 1), “Sometimes” (a score of 2), “Often” (a score of 3), “Most of the time” (a score of 4) and reverse scoring the results of positive answers. Then we merged these responses into a new indicator, namely depressive symptoms. Scores ranged from 8 to 32, and higher scores indicated greater severity of the depressive symptoms.

#### 2.2.2. Internet use

Internet use was measured in three aspects of this study: time spent on the internet, internet skills, and types of online activities. Time spent on the internet (hours) was calculated by adding up the online duration of mobile devices and computers in a day; the value ranges from 0 to 24. In CFPS in 2020, participants were asked “Do you play online games,” “Do you play online shopping,” “Do you watch short-form videos” and “Do you use WeChat?” in the past week, and we set the answer to “yes” = 1 and “no” = 0. Internet skills of older adults were measured by summing these 4 items, ranging from 0 to 4, with higher scores representing higher internet skills. By the above question, types of online activities are divided into four items: online games, online shopping, short-form videos, and WeChat. Respectively, each type is treated as a dichotomous variable.

#### 2.2.3. Covariates

Previous studies have found a set of variables associated with internet use and depressive symptoms (i.e., gender, age, education, marital status, monthly household income, place of residence, health status, and so on) (7, 18). What's more, Yu et al. (19) point out that lower memory ability at baseline was associated with worse depressive symptoms levels at follow-up. Therefore, to reduce the effects of confounding variables and provide stronger evidence of the association between internet use and depressive symptoms. We need to adjust for these variables, which include two main areas: sociodemographic variables and health status. The descriptive statistics of variables were shown in Table 1.

**Table 1:** Definition/codes of the potential confounding variables.

**Variable**	**Variable definition and assignment**
**Sociodemographic variables**	
Age	Continuous variable
Gender	0 = Male; 1 = Female
Marital status	0 = Single; 1 = Married
Education attainment	0 = Illiterate; 1 = Primary school; 2 = Junior middle school; 3 = Senior high school; 4 = College and above
Residency	0 = Rural; 1 = Urban
Number of family members	Continuous variable
Household income	Monthly Household income of people living in the household (logarithmically transformed)
**Health status**	
Self-rated health	According to respondents' self-rated physical health status. 0 = Excellent; 1 = Very good; 2 = Good; 3 = Fair; 4 = Poor
Chronic diseases	Ever had a doctor-diagnosed Chronic disease? 0 = No; 1 = Yes
Degree of memory	0 = Only remember one thing; 1 = Only remember a few; 2 = Remember half the things; 3 = Remember most things; 4 = R remember everything

### 2.3. Statistical analysis

Demographic characteristics of the sample were studied using descriptive analysis. The mean value (SD) is used for continuous variables and the frequency distribution is used for categorical variables.

We used multiple linear regression to analyze the relationship between internet use and depressive symptoms. A total of seven models were included, model one only included control variables. In model two, we added the time spent on the internet, and internet skills variables were added in model three. Models four to seven added types of online activities (online games, online shopping, short-form videos, and WeChat) in turn. Analyses were done using R (version 4.0.3; The R Foundation), and the level of significance was set at *P* < 0.05.

## 3. Results

### 3.1. Descriptive statistics

**Figure 1:**
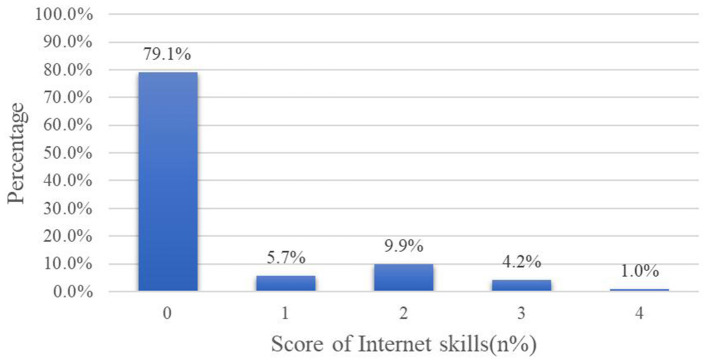
Percentage of different internet skills. N.B. The total percentage may not equal to 100 due to rounding.

**Figure 2:**
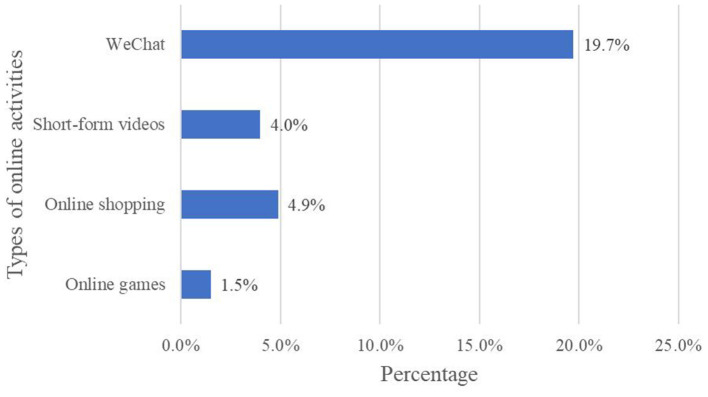
Percentage of using different types of online activities.

The basic characteristics of the sample and the distribution of the study variables by internet use for this study are shown in Table 2. Most of the samples (2,627/3,171, 82.8%) were married. There were slightly more males (51.7%) than females (48.3%), and the percentage of those living in rural areas is 50.3%. The average age of the sample was 68 (*SD* 5.8) years. There were 1294 samples who were illiterate. The mean monthly household income of the participants was ¥1958.33 (SD ¥2778.26; Mean US $288.41, SD US $409.17). The proportion of self-rated health as relatively good accounted for the most (1,149/3,171, 36.2%). There were 876 samples with chronic diseases. Regarding the degree of memory, there were 952 samples who only remembered one thing in the past week. The average online time was 0.4 (*SD* 1.3) h. As shown in Figure 1. The largest proportion of older adults with 0 Internet skills (2,507/3,170, 79.1%). While only 1.0% (33/3,170) of 4 Internet skills. As for different activities of internet use, the percentages of those using WeChat, short-form videos, online shopping, and online games were 19.7, 4.0, 4.9, and 1.5%, respectively (Figure 2). The average score for depression symptoms was 13.6 (*SD* 4.5).

**Table 2:** Sample characteristics of respondents.

**Variable**	**Class**	***n* (%)**	**Mean (SD)**	**Missing value (%)**
Gender	Female	1,532 (48.3)		0 (0)
	Male	1,639 (51.7)		
Age			68 (5.8)	0 (0)
Residency	Rural	1,588 (50.3)		14 (0.44)
	Urban	1,569 (49.7)		
Educational attainment	Illiterate	1,294 (40.8)		0 (0)
	Primary school	713 (22.5)		
	Junior high school	659 (20.8)		
	Senior high school	417 (13.2)		
	College and above	88 (2.8)		
Marital status	Single	544 (17.2)		0 (0)
	Married	2627 (82.8)		
Number of family members			3.8 (2.2)	3 (0.09)
Household income (¥^a^)			1958.33 (2778.26)	129 (4.07)
Self-rated health	Excellent	344 (10.8)		0 (0)
	Very good	346 (10.9)		
	Good	1149 (36.2)		
	Fair	556 (17.5)		
	Poor	776 (24.5)		
Chronic diseases	No	2,293 (72.4)		2 (0.06)
	Yes	876 (27.6)		
Degree of memory	Only remember one thing	952 (30.2)		18 (0.57)
	Only remember a few	580 (18.4)		
	Remember half the things	814 (25.8)		
	Remember most things	453 (14.4)		
	Remember everything	354 (11.2)		
Time spent on the internet			0.4 (1.3)	11 (0.35)
Depressive symptoms			13.6 (4.5)	0 (0)

a A currency exchange rate of US $1 = ¥6.79 is applicable.

N.B. The total percentage may not equal to 100 due to rounding.

### 3.2. Relationship between internet use and depressive symptoms

It shows the results of multiple linear regression models examining the associations between internet use and depression symptoms (Tables 3, 4). All models were statistically significant (for example, model one, *F* = 49.3, *P* < 0.001), and the independent variables could better explain the overall variation of depressive symptom scores (for example, Model one, *R*^2^ = 0.176). The results of model one showed that male (β = −0.63, *P* < 0.001), older (β = −0.05, *P* < 0.001), living in urban (β = −0.71, *P* < 0.001), married (β = −1.01, *P* < 0.001), higher education (junior high school: β = −0.58, *P* = 0.008; High school: β = −0.87, *P* < 0.001), better household income (β = −0.37, *P* < 0.001), better memory (β = −0.49, *P* < 0.001) were associated with lower depressive symptoms; Chronic disease (β = 0.6, *P* < 0.001) and lower self-rated health score (β = 0.84, *P* < 0.001) were associated with higher depressive symptoms. The results of model two and three showed that the higher internet skills (β = −0.42, *P* < 0.001), the lower the depressive symptoms; the longer the online time (β = 0.14, *P* = 0.03), and the more severe the depressive symptoms. The positive association between the time spent on the internet and the aggravation of depressive symptoms was more obvious when considering the skills (β = 0.31, *P* < 0.001).

**Table 3:** Effect of time spent on the internet and internet skills on depressive symptoms.

**Variable**	**Model 1**			**Model 2**			**Model 3**		
	β	***SE***	***P*** **-value**	β	***SE***	***P*** **-value**	β	***SE***	***P*** **-value**
Time spent on the internet				0.14	0.065	0.030	0.31	0.081	<0.001
Internet skills							−0.42	0.118	<0.001
Gender (reference: female)									
Male	−0.63	0.161	<0.001	−0.63	0.162	<0.001	−0.64	0.161	<0.001
Age	−0.05	0.014	<0.001	−0.05	0.014	0.001	−0.05	0.014	<0.001
Residency (reference: rural)									
Urban	−0.71	0.166	<0.001	−0.75	0.167	<0.001	−0.72	0.167	<0.001
Marital status (reference: single)									
Married	−1.01	0.212	<0.001	−1.00	0.213	<0.001	−1.00	0.212	<0.001
Educational attainment (reference: illiterate)									
Primary school	−0.38	0.202	0.061	−0.39	0.203	0.054	−0.37	0.202	0.068
Junior high school	−0.58	0.218	0.008	−0.62	0.220	0.005	−0.55	0.220	0.012
Senior high school	−0.87	0.262	<0.001	−0.99	0.269	<0.001	−0.84	0.272	0.002
College and above	−0.61	0.496	0.221	−0.72	0.504	0.153	−0.57	0.505	0.259
Number of family members	−0.06	0.034	0.067	−0.06	0.034	0.097	−0.06	0.034	0.079
Chronic diseases (reference: no)									
Yes	0.60	0.175	<0.001	0.59	0.176	<0.001	0.60	0.175	<0.001
Household income	−0.37	0.081	<0.001	−0.40	0.082	<0.001	−0.38	0.082	<0.001
Self–rated health	0.84	0.063	<0.001	0.84	0.063	<0.001	0.83	0.063	<0.001
Degree of memory	−0.49	0.058	<0.001	−0.49	0.059	<0.001	−0.48	0.059	<0.001
*R^2^*	0.176			0.176			0.18		

**Table 4:** Effects of types of online activities on depressive symptoms in older adults.

**Variable**	**Model 4**			**Model 5**			**Model 6**			**Model 7**		
	β	***SE***	***P*** **-value**	β	***SE***	***P*** **-value**	β	***SE***	***P*** **-value**	β	***SE***	***P*** **-value**
Online games (reference: no)												
Yes	0.54	0.690	0.434									
Online shopping (reference: no)												
Yes				−0.27	0.470	0.570						
Short–form videos (reference: no)												
Yes							1.24	0.069	0.049			
WeChat (reference: no)												
Yes										−0.96	0.462	0.040
Time spent on the internet	0.35	0.084	<0.001	0.31	0.083	<0.001	0.38	0.109	<0.001	0.33	0.082	<0.001
Internet skills	−0.50	0.133	<0.001	−0.43	0.143	0.003	−0.98	0.250	<0.001	−0.06	0.205	0.758
Gender (reference: female)												
Male	−0.64	0.163	<0.001	−0.63	0.161	<0.001	−0.65	0.172	<0.001	−0.65	0.161	<0.001
Age	−0.05	0.014	<0.001	−0.05	0.143	<0.001	−0.05	0.015	0.001	−0.05	0.014	<0.001
Residency (reference: rural)												
Urban	−0.72	0.168	<0.001	−0.72	0.166	<0.001	−0.79	0.176	<0.001	−0.72	0.167	<0.001
Marital status (reference: single)												
Married	−1.00	0.214	<0.001	−0.95	0.212	<0.001	−0.93	0.225	<0.001	−0.99	0.212	<0.001
Educational attainment (reference: illiterate)												
Primary school	−0.35	0.203	0.081	−0.36	0.202	0.071	−0.29	0.210	0.169	−0.36	0.202	0.079
Junior high school	−0.53	0.222	0.016	−0.55	0.220	0.012	−0.59	0.234	0.012	−0.52	0.221	0.019
Senior high school	−0.79	0.276	0.004	−0.91	0.272	<0.001	−0.71	0.300	0.018	−0.78	0.273	0.004
College and above	−0.50	0.521	0.341	−0.50	0.508	0.323	−0.09	0.570	0.869	−0.50	0.506	0.323
Number of family members	−0.06	0.035	0.077	−0.06	0.034	0.104	−0.07	0.036	0.069	−0.06	0.344	0.071
Chronic diseases (reference: no)												
Yes	0.60	0.177	<0.001	0.62	0.175	<0.001	0.69	0.167	<0.001	0.62	0.175	<0.001
Household income	−0.36	0.083	<0.001	−0.37	0.082	<0.001	−0.33	0.087	<0.001	−0.36	0.082	<0.001
Self–rated health	0.84	0.064	<0.001	0.81	0.063	<0.001	0.81	0.067	<0.001	0.83	0.063	<0.001
Degree of memory	−0.48	0.059	<0.001	−0.49	0.059	<0.001	−0.49	0.623	<0.001	−0.48	0.059	<0.001
*R^2^*	0.179			0.182			0.174			0.181		

Taking into account control variables, time spent on the internet, and internet skills. The effect of different types of online activities on depressive symptoms needs to be considered (Table 4). The results showed that watching short-form videos (β = 1.24, *P* = 0.049) was associated with higher depressive symptoms, and using WeChat (β = −0.96, *P* = 0.040) was associated with lower depressive symptoms, while playing online games and online shopping had no significant effect.

## 4. Discussion

### 4.1. Principal findings

Taking into account the rich internet use of older adults in China and the phased characteristics of internet use, this study aims to reveal the complex association between internet use and depressive symptoms in older adults by using CFPS. The main conclusions are as follows: First, longer time of internet use is associated with higher depressive symptoms. Second, higher internet skills are associated with lower depressive symptoms. Third, watching short-form videos is associated with higher depressive symptoms, and using WeChat is associated with lower depressive symptoms. In general, the effect of internet use on depression symptoms in older adults is a double-edged sword. Previous studies have generally been optimistic about information technology use, but this study reveals the dark side of information technology use.

### 4.2. Possible explanations and relations to previous studies

The present study showed that being male, older, living in an urban, married, having higher education, and having better household income were associated with lower depressive symptoms. Our finding is consistent with the previous study that demographic-related variables are significantly related to depressive symptoms (7, 18). Lower levels of memory, chronic illness, and lower self-health scores were associated with higher depressive symptoms. Previous literature suggests older adults may experience an increase in depressed affect due to poor memory function (20). Yuan (7) pointed out depression symptoms sum scores increase with the diagnosis of chronic diseases.

The longer the Internet use, the worse the depressive symptoms. Our finding is consistent with a previous study on adolescents (21–23). Wu et al. (23) found adolescents spending more time online had a higher risk of experiencing depression symptoms. An increase of 1 h in average internet usage was related to an increase of 0.69 units in the severity of depressive symptoms (22). Excessive internet use can negatively affect individuals' lives (24–26), which may be explained by the fact that excessive internet use disconnects older adults from fundamental social interactions and activities, thereby impairing their mental health (26).

Our results found that older adults who have higher internet skills generally have lower depressive symptoms. The finding is consistent with previous studies showing the protective effects of higher internet skills on depressive symptoms (27) and the psychological wellbeing of older adults. By mastering internet skills, older adults can obtain required health information (e.g., using a medical website to learn about a health condition), social engagement (video conferencing to see distant relatives, sending text messages to friends or video chatting to buffer against loneliness) and so on to maintain mental health (28, 29).

Playing online games and online shopping have no significant relationship with depressive symptoms in older adults. However, Numerous studies have shown that involvement in leisure activities (e.g., playing online games) has a significant impact on older adults' psychological, improving their quality of life (30, 31). These possible explanations are that this study adopted a nationally representative sample, showing that the proportion of older adults playing online games and online shopping is small; only 1.5% of the sample played online games, and 4.9% shopped online. Besides, this study carefully controlled for time spent on the internet and internet skills effects. Online gaming and online shopping did not affect depression in older adults, at least at this stage.

As for watching short-form videos, it can aggravate depressive symptoms, which is consistent with Perlis et al. (32), who found TikTok usage was significantly associated with a greater risk of an increase in self-reported depressive symptoms. This finding was also consistent with practical observation. Considering the time spent on the internet and internet skills, using short-form videos by older adults may exacerbate depressive symptoms. The reason for this is that briefly stimulating, low-quality short videos in the current internet market are harmful to the mental health of older adults.

Social media use in older adults can potentially improve mental health in older adults (33–35). Qu et al. (34) used a multilevel logistic regression model for data analysis and found a significant reduction in the prevalence of depressive symptoms associated with WeChat use after adjusting for all possible covariates; the results of a review also found that online social networks can be used as a potential social therapy to alleviate depressive symptoms (35). Our findings reinforce previous findings, providing evidence that WeChat use is associated with lower CES-D scores, suggesting that social media use may be beneficial for depressive symptoms. For older populations, contacting family and friends when they were geographically separated and entering intergenerational communication with younger family members are much more important needs (36). Therefore, using WeChat can increase the chances of older adults' support of social exchange, reduce social isolation and loneliness, and thus improve the symptoms of depression.

### 4.3. Limitations

The findings should be interpreted with caution because of the following limitations. First, the data are cross-sectional design; it was impossible to conclude the causal relationship between internet use and depressive symptoms, and subsequent studies should use longitudinal analysis or randomized controlled trials to test the causal effect. Second, depressive symptoms used for the analyses were based on a self-report questionnaire, increasing the possibility of recall bias. Besides, the score of the CES-D scale can evaluate the depressive symptoms of older adults, which cannot be used to judge whether older adults suffer from depression. Third, owing to the limitations of the questionnaires, the analysis only included four types of online activities. The specific contribution of many common online activities to overall depressive symptoms during aging remains unclear. Fourth, the data reanalysis scheme inevitably has limitations in variable measurement. Although there is no clear standard on how to quantify internet skills, the variables selected and constructed in this study are still limited due to the limitations of the data itself, and subsequent studies should consider more accurate and in-depth measurement and analysis.

### 4.4. Implications for public health

This study finds that information technology is a double-edged sword, as it contributes to successful aging on the one hand and brings back harmful effects on the other. Therefore, how to let older adults thoroughly enjoy digital benefits and a better quality of life in old age while avoiding the dark side of the Internet is an essential issue that the whole society should discuss and solve together in the digital age. From the public perspective, the government, community, and families should participate together: the government should formulate relevant policies to provide institutional protection for older adults to use the Internet; the community should strengthen internet safety propaganda and actively organize internet use training; families should participate in improving internet use literacy of old persons, pay attention to internet use behavior of older adults, and reduce the infringement of certain internet use behaviors on the family network.

## Data availability statement

The original contributions presented in the study are included in the article/supplementary material, further inquiries can be directed to the corresponding author.

## Author contributions

AM and SY planned the study, performed the data analysis, and wrote the paper. ZL provided comments on the changes. All authors contributed to the article and approved the submitted version.
